# Vav1 Fine Tunes p53 Control of Apoptosis versus Proliferation in Breast Cancer

**DOI:** 10.1371/journal.pone.0054321

**Published:** 2013-01-14

**Authors:** Shulamit Sebban, Marganit Farago, Dan Gashai, Lena Ilan, Eli Pikarsky, Ittai Ben-Porath, Shulamit Katzav

**Affiliations:** 1 Departement of Developmental Biology and Cancer Research, Institute for Medical Research Israel-Canada, Hadassah Medical School - Hebrew University, Jerusalem, Israel; 2 Deaprtment of Immunology & Cancer Research and Department of Pathology, Institute for Medical Research Israel-Canada, Hadassah Medical School - Hebrew University, Jerusalem, Israel; Vanderbilt University Medical Center, United States of America

## Abstract

Vav1 functions as a signal transducer protein in the hematopoietic system, where it is exclusively expressed. Vav1 was recently implicated in several human cancers, including lung, pancreatic and neuroblasoma. In this study, we analyzed the expression and function of Vav1 in human breast tumors and breast cancer cell lines. Immunohistochemical analysis of primary human breast carcinomas indicated that Vav1 is expressed in 62% of 65 tumors tested and is correlated positively with estrogen receptor expression. Based on published gene profiling of 50 breast cancer cell lines, several Vav1-expressing cell lines were identified. RT-PCR confirmed Vav1 mRNA expression in several of these cell lines, yet no detectable levels of Vav1 protein were observed due to cbl-c proteasomal degradation. We used two of these lines, MCF-7 (Vav1 mRNA negative) and AU565 (Vav1 mRNA positive), to explore the effect of Vav1 expression on breast cell phenotype and function. Vav1 expression had opposite effects on function in these two lines: it reduced proliferation and enhanced cell death in MCF-7 cells but enhanced proliferation in AU565 cells. Consistent with these findings, transcriptome analysis revealed an increase in expression of proliferation-related genes in Vav1-expressing AU565 cells compared to controls, and an increase in apoptosis-related genes in Vav1-expressing MCF-7 cells compared with controls. TUNEL and γ-H2AX foci assays confirmed that expression of Vav1 increased apoptosis in MCF-7 cells but not AU565 cells and shRNA experiments revealed that p53 is required for this pro-apoptotic effect of Vav1 in these cells. These results highlight for the first time the potential role of Vav1 as an oncogenic stress activator in cancer and the p53 dependence of its pro-apoptotic effect in breast cells.

## Introduction

The physiological function of Vav1 is restricted to the hematopoietic system [1], where it plays a critical role in the development and activation of T-cells. Following stimulation of the TCR, Vav1 is phosphorylated at N-terminal tyrosine amino acid residues, and this upregulates its Guanine Nucleotide Exchange Factor (GEF) activity for specific Rho/RacGTPases, leading to actin cytoskeletal reorganization [2]. Vav1 also regulates calcium, ERK-MAP kinase, NFAT and NF- κB signaling pathways in B and T-cells [3], [4]. Recent studies revealed that wild-type Vav1, which is normally tightly restricted to hematopoietic cells, is expressed in several human tumor malignancies, suggesting that it has a role in human cancer.

The involvement of wild type Vav1 in human tumors was first demonstrated in the neuroblastoma SK-N-MC cell line [5]. A subsequent screen of 42 primary human neuroblastomas revealed that the majority expressed Vav1. Wild-type Vav1 was also identified in more than 50% of 95-pancreatic ductal adenocarcinoma (PDA) specimens examined and in several PDA cell lines [6]. Patients with Vav1-positive tumors had a worse prognosis than patients with Vav1-negative tumors [6]. Aberrant expression of Vav1 was also found in over 40% of human primary lung cancers and lung cancer cell lines examined [7] and in melanoma tissue sections and cell lines [8]. Expression of Vav1 was also shown in hematological malignancies such as B cell chronic lymphocytic leukemia (B-CLL), occurring primarily in B-CLL patients with 13q chromosomal deletions [9]. Depletion of Vav1 expression in pancreatic and lung cancer cell lines reduced colony formation in soft agar and tumor size in nude mice. This effect of Vav1 silencing was observed even in the presence of mutant K-Ras, demonstrating the critical role of Vav1 in tumor development [6], [7].

Vav1 might contribute to malignancy by activating signaling cascades through its GEF activity, resulting in cytoskeletal reorganization and transcription 10–12. Despite its physiological restriction to hematopoietic cells, Vav1 can be phosphorylated on tyrosine residues in cells of other tissue origins following stimulation of growth factor receptors such as EGFR [13], platelet derived growth factor receptor (PDGFR) [14], and the Nerve Growth Factor (NGF) receptor, trk [15]. The additional Vav1-triggered signaling may overwhelm cellular control mechanisms and promote transformation.

To increase our understanding of Vav1 activity and regulation in human cancers, we analyzed the involvement of Vav1 in human breast cancer. In this study, we show that Vav1 is expressed in the majority of breast carcinomas and that its ectopic expression in breast cancer cell lines can induce significant changes in these cells, causing either transformation or cell death.

## Materials and Methods

### Human Breast Tissue Array

Human breast paraffin tissue array (http://www.biochain.com/biochain/datasheet/Z7020004-B410017.pdf) was purchased (Biochain, CA, USA) and treated according to manufacturer's instructions.

### Immunohistochemistry

Immunostainings were performed with anti-Vav1 mAbs, 1∶10,000 (Upstate Biotechnology, NY, USA) and one with no antibody using the labeled streptavidin biotin (LAB-SA) technique (Zymed Laboratories, CA, USA) according to the manufacturer's instructions. Staining was evaluated by a board certified pathologist (E.P) and was quantified as described [7].

### Gene Array

mRNA was isolated from cells using the RNeasy mini kit (QIAGEN, Germany), and samples were subjected to GeneChip® Human Exon 1.0 ST Array (Affymetrix, CA, USA). Each sample was composed of a mixture of three independent mRNA isolations. Data was read and RMA normalized using Partek Genomic Suite 6.6. Statistical testing for significant genes and clustering used this package, in addition to dedicated packages written in Matlab R2011A.

### RT-PCR

Total RNA and reverse transcription of Vav1 and GAPDH was performed as previously described [7].

### Quantitative Real-time PCR

Total RNA and cDNAs from cell lines were prepared as above. Detection of Vav1 was performed using cyber green PCR master mix (Tamar, Jerusalem, Israel) and the required primers (Table S1). Analysis was performed using the ABI Prism 7300 real-time PCR technology (Applied Biosystems, CA, USA). Three independent experiments were performed, each in triplicate.

### Cell Culture, Cell Stimulation and Vav1 Expression

Jurkat (acute T cell leukemia, kindly given to us by Dr. Weiss [16]), U937 (monocytes, histiocytic lymphoma [17]), H358 (bronchioalveolar Non-Small Lung Carcinoma, kindly given to us by Drs. Gazdar and Minna [18]), AU565 [19] and SK-BR-3 [20] were grown in RPMI medium (Sigma); and MCF-7 cells [21] were grown in Dulbecco's modified Eagle's medium (DMEM) (Sigma). All media was supplemented with 10% Fetal Bovine Serum (FBS), Penicillin-Streptomycin and L-Glutamine (Biological Industries, Israel) and cells were maintained at 37°C with 5% CO_2_. For stimulation with CSF1 or EGF, cells were grown to sub-confluence, starved in serum-free medium for 48 h and treated with medium containing 50 ng/ml human CSF1 (Peprotech, NJ, USA) or 100 ng/ml human EGF (Cytolab, Rehovot, Israel) for 5, 15 and 30 min at 37°C. For expression of Vav1, cDNA encoding the entire Vav1 coding region was generated by PCR and inserted by in-frame cloning into the vector pcDNA6 (Invitrogen, NY, USA) at two *Bst*BI restriction sites. AU565 and MCF-7 cells were stably transfected with either 2 µg of Vav1-pcDNA6 or 2 µg of empty pcDNA6 vector, using the jetPEI® transfection reagent (Polyplus, CA, USA). Transfected cells were selected using 7 µg/ml blasticidine (Invitrogen, NY, USA). For luciferase assay, AU565 cells were transiently transfected with pGL3-Vav1 or pGL3 vector using the jetPEI® transfection reagent.

### Luciferase Reporter Assay

Luciferase reporter assay was performed with Dual- Luciferase Reporter System (Promega, USA) using a Luminometer Mithras (Berthold Technologies, Germany) as described [22].

### Quantitative Real-time PCR

Detection of Vav1 on cDNAs (see above) was performed using cyber green PCR master mix (Tamar, Jerusalem, Israel) and the required primers (Table S1). Analysis was performed using the ABI Prism 7300 real-time PCR technology (Applied Biosystems, CA). Three independent experiments were performed in triplicates.

### Immunoprecipitation and Immunoblotting

Cell lysis, immunoprecipitation, and immunoblotting procedures were performed as described [7] by using the antibodies outlined in Table S2.

### Immunofluorescence

Immunofluorescence was performed as described [23] using anti-Vav1 mAbs and secondary AlexaFluor-647 anti-mouse IgG1. Hoechst dye was used for nuclei staining.

### GEF Activity of Vav1

Cells (2.5×10^6^) were transfected with 1 µg of FLAG epitope-tagged Rac plasmid as indicated. Rac activation was analyzed using a GST-Pak1 pull-down assay [24].

### MTT *Cell Proliferation Assay*

Cells were grown in 6 well plates to sub-confluence and then starved for 96 h. During this time period, 0.1 mg/ml of MTT (3-(4,5-Dimethylthiazol-2-yl)-2,5-diphenyltetrazolium bromide) in dimethyl sulfoxide was added to 3 wells of each cell type, starting at 0 h, in 24 h intervals. Absorbance was quantified at 540 nm.

### Soft Agar Colony Formation Assay

The soft agar assay was carried out as previously described [7]. Three independent experiments were performed, each one in triplicate.

### shRNA

Cells were infected with pLKO-based (Open Biosystems) lentiviral vector with or without the human TP53, CBLC or VAV1- shRNA encoding sequences (Table S1). Transfected cells were selected with puromycin.

### Proteasome Inhibition

Proteasome inhibition was carried out using 10 µM MG132 (carbobenzoxy-Leu-Leu-leucinal) inhibitor (AGC Scientific, CA, USA). Cells were lysed after 4 hr incubation and subjected to immunoblotting as described above.

### TUNEL Assay

*In Situ* Cell Death Detection Kit was purchased (Roche Applied Science, USA) and used according to manufacturer's instructions.

### Statistical Analysis

Unpaired Student's *t*-test was used to evaluate statistical significance.

## Results

### Vav1 is Expressed in the Majority of Human Breast Tumors

**Figure 1:**
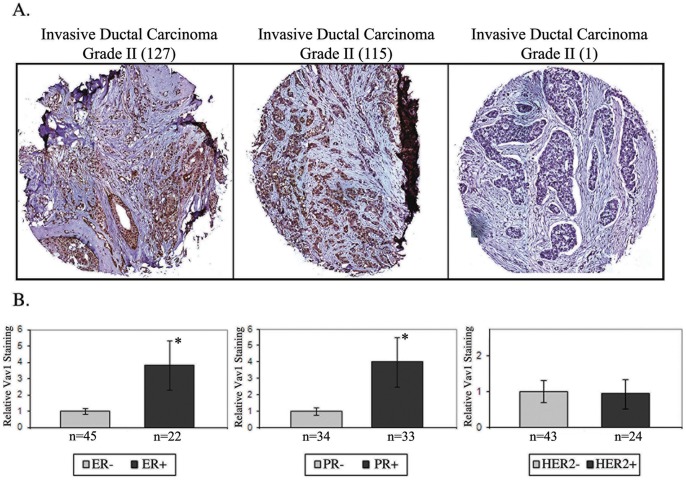
Vav1 is expressed in the majority of human breast tumors. An array of 70 specimens of human breast tumors was stained with anti-Vav1 monoclonal antibody. (A) Anti-Vav1 staining of three representative invasive ductal carcinoma grade II tumor specimens. (B) Vav1 expression correlated positively with expression of estrogen and progesterone receptors, but not with HER2 expression. Mean Vav1 expression values of groups were compared using the Unpaired Student's *t*-test, and normalized to each other. (*) indicates p<0.02 value.

We assessed Vav1 expression using a commercial human breast tissue array containing 70 cases of reactive, premalignant and malignant tumors of various grades and stages and five normal controls in duplicates. 32% of tumors were estrogen receptor (ER) positive, 27% were progesterone receptor (PR) positive (including 15% that were both ER and PR positive) and 50% were HER2 positive. Immunohistochemical analysis against Vav1 was performed, and Vav1 staining was quantified using an automated robotic image analysis system. Five lobular invasive carcinoma samples were excluded due to small case number. Vav1 was expressed at varying intensities in 40 of the 65 remaining tumors (62%) (Fig. 1A and Table S3) and was correlated positively with expression of ER and PR but not with HER2 expression (Fig. 1B).

### Expression of Vav1 Protein in Breast Cancer Cell Lines is Regulated by Cbl-c Ubiquitin Ligase

**Figure 2:**
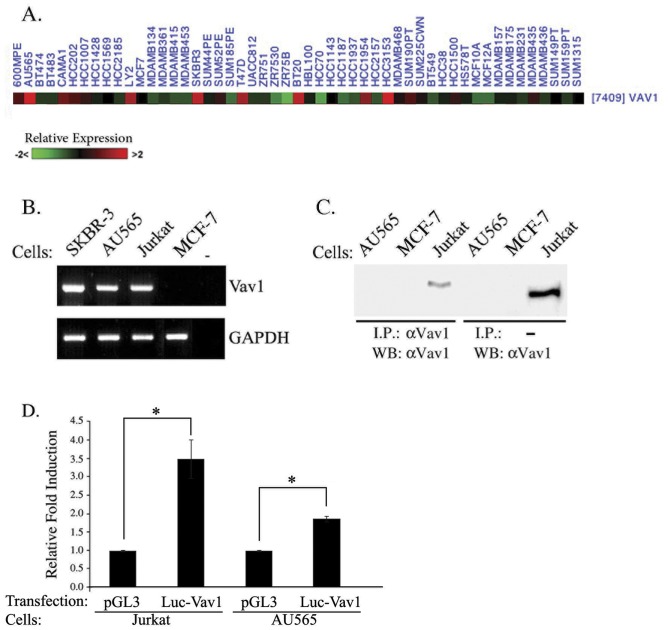
Expression of Vav1 in breast cancer cell lines. (A) Expression levels of Vav1 in a series of 50 breast cancer cell lines relative to the mean expression of each gene across cell lines. Data were derived from a previously published gene expression profiling study [25]. Color intensity indicates the relative level of Vav1 expression: red- greater than mean, green- less than mean, black- mean expression. (B) Vav1 mRNA expression in several cell lines (AU565, MCF-7 and SKBR3 breast cancer cells and Jurkat T cells) was analyzed by RT-PCR. (C) Total lysates (right) and immunoprecipitates with polyclonal anti-Vav1 antibody (left) from AU565 and MCF-7 cells were separated on SDS-PAGE and immunoblotted with monoclonal anti-Vav1 antibody. (D) Wild-type luciferase reporter gene (Le2) was transfected into the cell lines and luciferase activity was measured 24 hr later. Data show luciferase activity normalized to *Renilla* transfection efficiency control and calculated relative to the luciferase activity of an empty vector expression, pGL3, in each line. Jurkat T cells were used as a positive control for Vav1-expressing cells. Data are the mean of 5 experiments. Unpaired Student's *t*-test was used. (*) indicates p<0.03 value.

We determined the relative expression levels of Vav1 in a series of 50 breast cancer cell lines using data from a gene expression profiling study [25]. This analysis identified 8 cell lines with relatively high Vav1 mRNA expression and 15 additional lines with intermediate levels of Vav1 expression (Fig. 2A). RT-PCR indicated Vav1 mRNA expression in five out of 13 cell lines: AU565, SK-BR-3, MDA-MB-468, T47D and HCC1954 (Table S4; Fig. 2B). Surprisingly, protein analysis detected no or very low levels of Vav1 protein in these cell lines (Fig. 2C; data not shown). To assess the transcriptional activity of the *vav1* promoter in AU565 cells, we transfected these cells as well as Jurkat T cells with a pGL3-*vav*1 reporter construct containing the minimal regulatory sequences from the *vav*1 proximal promoter region upstream of a luciferase reporter gene [22]. Luciferase expression in AU565 cells transfected with pGL3-*vav1* was two-fold higher than in cells transfected with the pGL3 vector control (Fig. 2D). These data suggest the Vav1 promoter is active, consistent with the presence of Vav1 mRNA in these cells.

**Figure 3:**
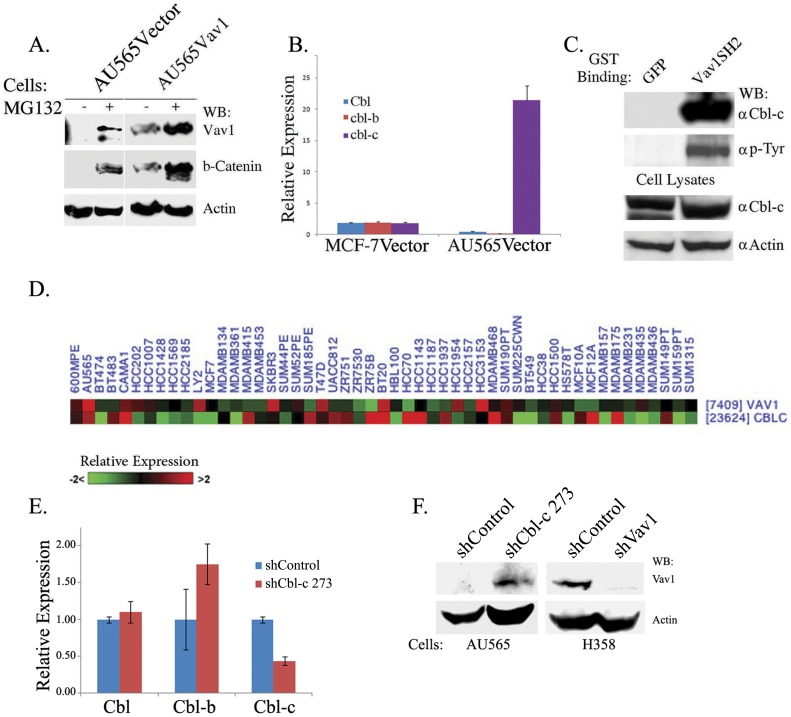
Vav1 is regulated by the Cbl-c ubiquitin ligase in breast cancer cells. (A) AU565Vector and AU565Vav1 were treated with10 mM of the proteasome inhibitor MG132 for four hours or left untreated. Cell lysates were subjected to immunoblotting with anti-Vav1 antibody. Anti β-catenin antibody was used as a control for an unstable protein with a short half-life. Anti-actin antibody served as a loading control. (B) The expression of Cbl isoforms (Cbl, Cbl-b and Cbl-c) was analyzed by Real-Time PCR in MCF-7Vector and AU565Vector cells. (C) Lysates of AU565Vav1 cells were incubated with bacterial GST fusion proteins expressing the SH2 domain of Vav1 (GST-Vav1SH2) or GFP (GST-GFP), and then immobilized on glutathione-Sepharose beads. Bound proteins were resolved on SDS-PAGE gels and immunoblotted with anti-Cbl-c or anti-pTyr antibodies. As loading control, total cell lysates were immunoblotted with anti-Cbl-c and actin antibodies. (D) Vav1 and Cbl-c expression in 50 breast cancer -originated cell lines using the same gene array as in Figure 2A. Red: greater than mean expression, Green: less than expression, Black: mean expression. (E) Cbl-c is silenced in AU565 cells following treatment with Cbl-c shRNA. Cells were infected with viral vectors containing shRNA for Cbl-c (shCbl-c 273) or control validated (shControl). cDNA was subjected to real time PCR analysis using primers against Cbl-c, Cbl and Cbl-b. GAPDH was used as a control. (F) Lysates from AU565 cells infected with shCbl-c 273 or control shScrambled were subjected to immunoblotting with anti-Vav1 antibody. As a control for Vav1 expression, lysates from H358 lung cancer cells infected with shVav1 or shControl were used. Anti-actin was used as loading control.

The fact that we do not detect Vav1 protein in cells with active *vav1* transcription and relatively high Vav1 mRNA expression might stem from rapid degradation of the protein by the proteasome following translation. Therefore, we incubated AU565 cells stably transfected with either an empty vector (AU565Vector) or with Vav1 expression vector (AU565Vav1) with the proteasome inhibitor MG132 for four hours. Western blotting revealed Vav1 protein accumulation in both control and Vav1-transfected cells (Fig. 3A).

Vav1 was shown to undergo Cbl-dependent ubiquitination in T-cells [26], [27]; therefore, we analyzed the expression of the three Cbl family members, Cbl, Cbl-b and Cbl-c, in MCF-7Vector (MCF-7 cells stably transfected with an empty vector) and AU565Vector cells. While Cbl and Cbl-b were expressed at low levels in MCF-7Vector cells and at lower levels in AU565Vector cells, Cbl-c was 20-fold higher in AU565Vector compared with MCF-7Vector cells (Fig. 3B). Similar results were obtained with the cells stably expressing Vav1 (MCF-7Vav1 and AU565Vav1, data not shown). To analyze whether Vav1 and Cbl-c physically associate in these cells, we incubated lysates of AU565Vav1 cells with a protein containing the SH2 domain of Vav1 fused to gluthatione-S-transferase (Vav1SH2-GST). Proteins bound to the Vav1SH2-GST fusion protein or to a control GFP-GST fusion protein were separated and Western blotted with antibodies against Cbl-c. Our results clearly demonstrate a specific association between the Vav1SH2 domain and Cbl-c (Fig. 3C). Since a Vav1SH2-associated protein that has the exact molecular weight as the Vav1SH2-CBl-c protein was recognized by anti-phospho-tyrosine antibodies, we assume that the Vav1SH2-Cbl-c association is tyrosine dependent (Fig. 3C). High Cbl-c expression was found in 6 of the 8 lines with high Vav1 expression (Fig. 3D; Table S4). Knock-down of Cbl-c in AU565 cells (Fig. 3E) resulted in the appearance of Vav1 expression in these cells (Fig. 3F), attesting to the control of Vav1 expression by Cbl-c.

### Vav1 as a Signal Transducer in Breast Cancer Cell Lines

Vav1 is found to be expressed in a large proportion of human breast tumors illustrating its potential huge importance in breast cancer biology. Accordingly, we find mRNA expression of Vav1 is many breast cancer cell lines (Fig. 2A, Table S4); surprisingly we find little or no Vav1 protein mainly due to degradation by Cbl-c. This suggests the existence of complex mechanisms or regulation of Vav1 expression in breast tumors *in vivo*. To overcome this hurdle for studying the functional role of Vav1 in human breast cancer, we overexpressed Vav1 in two breast cancer cell lines, AU565 and MCF-7, achieving Vav1 protein levels which on immunohistochemical assay are similar to those present in primary human tumors.

**Figure 4:**
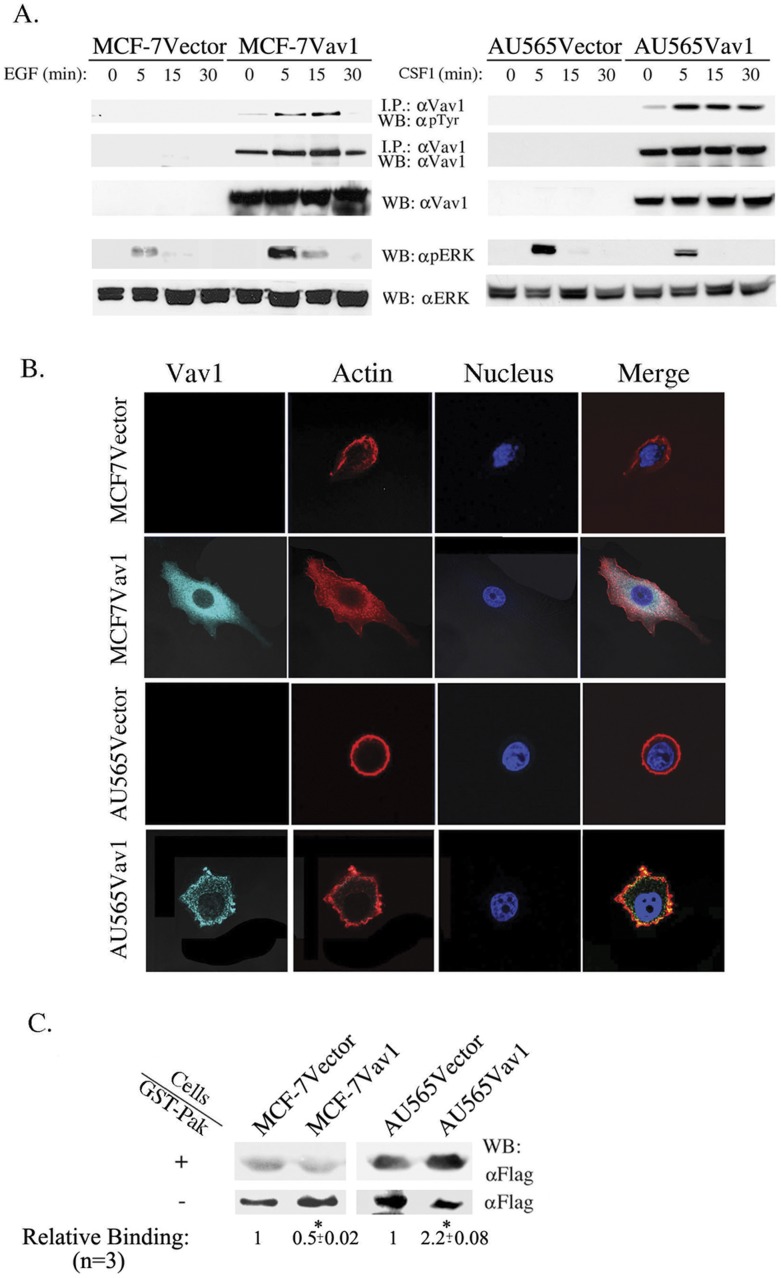
Vav1 as a signal transducer protein in breast cancer cells. (A) MCF-7Vector, MCF-7Vav1, AU565Vector and AU565Vav1 were stimulated with EGF or CSF1, respectively, for various times as indicated. Cell lysates were immunoprecipitated with anti-Vav1 antibody and then immunoblotted with either anti-Vav1 antibody or anti- pTyr antibody (top 2 immunoblots). In addition, total cell lysates were separated on SDS-PAGE and immunoblotted with anti-Vav1, anti-pERK or anti-ERK antibodies (lower 3 immunoblots). (B) Immunofluorescence of 145 MCF-7Vector, 176 MCF-7Vav1, 355 AU565Vector and 174 AU565Vav1 with anti-Vav1 antibody. Actin filaments were detected by phalloidin and nuclei were stained with Hoechst. The difference in morphology between MCF-7Vav1, AU565Vav1 and their corresponding control cells were highly significant (two-tailored pValue; 0.0002 and 0.0024 respectively). Representative photographs taken with a Zeiss LSM 710 confocal microscope and analyzed by the ZEN 2010 program are shown. (C) MCF-7Vector, MCF-7Vav1, AU565Vector and AU565Vav1 were transiently transfected with Flag-Rac. 48 hours later, cell lysates were incubated with GST–PAK bacterial fusion proteins immobilized on glutathione sepharose beads. Bound proteins (+) and unbound proteins (−) were separated on SDS–PAGE and immunoblotted with anti-Flag mAbs. Numbers indicate mean (+/− S.E.) relative binding from three different experiments. Unpaired Student's *t*-test was used. (*) indicates p<0.05 value.

To study whether ectopically expressed Vav1 in breast cancer cells is functionally active, we stimulated MCF-7Vector and MCF-7Vav1 cells with EGF and AU565Vector and AU565Vav1 with CSF1 for various time intervals. MCF-7 cells are ER-positive, HER-2-negative, EGFR positive and express wild-type p53, while AU565 cells are ER-negative, HER-2-positive, EGFR negative and express mutant p53. In addition, AU565 cells express the CSF1 receptor (data not shown). By using an approach employed by us previously [28], we have demonstrated that tyrosine phosphorylation of Vav1 in EGF-treated MCF-7Vav1 and CSF1-treated AU565Vav1 cells. While phosphorylation of Vav1 in MCF-7Vav1 and AU565Vav1 cells was noted beginning 5 minutes following stimulation, it lasted a longer period in AU565Vav1 cells (Fig. 4A).

Activated Vav1 was previously shown to elevate ERK phosphorylation in some cells and not others [29]–[31]. ERK phosphorylation was significantly enhanced in MCF-7Vav1 cells compared to MCF-7Vector, while the level of ERK phosphorylation was similar in AU565Vav1 and AU565Vector cells (Fig. 4A).

Recent studies in pancreatic cancer [6] and lung cancer [7] cells showed that ectopically expressed Vav1 acts as an upstream activator of Rac1, RhoA and possibly Cdc42 signaling pathways in response to extracellular stimulation, leading to cytoskeleton changes in cancer cells. To examine cytoskeletal structure, we analyzed actin organization in Vav1- and control-transfected cells of both cell lines by immunofluorescence. MCF-7Vav1 cells were more flattened than control MCF-7Vector cells (Fig. 4B). AU565Vav1 cells lost their round shape and formed lamellipodia (Fig. 4B). Since Vav1 activates Rac1 in immune cells, we examined Rac1-GTP activation in the Vav1-expressing breast cancer cell lines. MCF-7Vav1 and AU565Vav1 and control cells were transiently transfected with Flag tagged-Rac1. Cell lysates were incubated with control GST-GFP or with GST–PAK (p21 activated kinase 1), a fusion of GST with the Rac/Cdc42 binding domain (PBD) of human PAK [24]. As expected, in AU565 cells, expression of Vav1 induced activation of Rac as evident by increased binding to GST-PAK. However, In MCF-7 cells, similar expression downmodulated Rac activity (Fig. 4C). Importantly, basal activation of Rac1 was greater in AU565 cells than in MCF-7 cells (Fig. 4C).

### Vav1 Expression has an Antagonizing Effect on Proliferation and Tumorigenicity in AU565 and MCF-7 Cells

Although Vav1 expression affected cytoskeletal organization in both MCF-7Vav1 and AU565Vav1 cells, Rac1 activity was elevated only in AU565Vav1 cells. Elevated Rac1 activity may also be associated with changes in other cell functions, including anti-apoptotic pathways and regulation of gene expression [32]. We searched for additional Vav1-related biological differences between AU565Vav1 and MCF-7Vav1, beginning by analyzing cell proliferation using MTT and soft agar colony formation assays.

**Figure 5:**
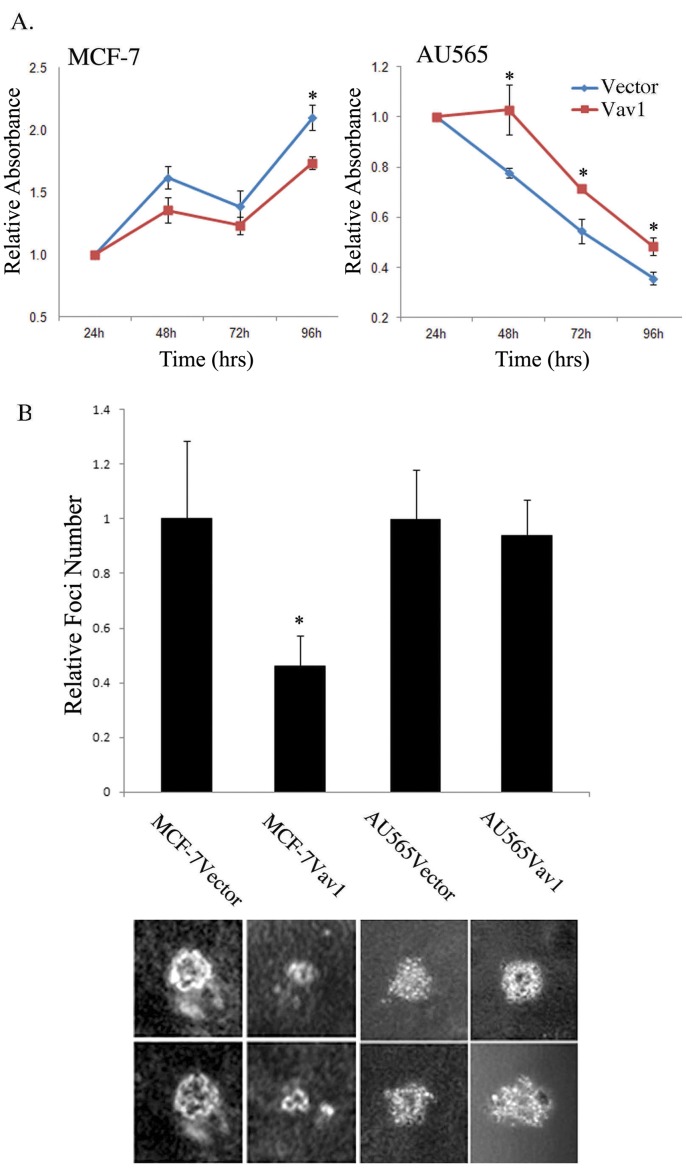
Vav1 expression has an antagonistic effect on proliferation and tumorigenicity in AU565 and MCF-7 cells. (A) MTT assay of MCF-7Vector, MCF-7Vav1, AU565Vector and AU565Vav1 cells. Sub-confluent cells were starved for 96 hr and proliferation was measured by MTT assay. Data show mean absorbance at 550 nm (relative to 24 hour value) of 3 independent experiments. (*) indicates p<0.05 value (B) Soft agar colony formation assay. MCF-7Vector, MCF-7Vav1, AU565Vector and AU565Vav1 were suspended in RPMI medium containing 0.3% agar and 10% calf serum, and plated onto a bottom layer containing 0.8% agar. Cells were plated in triplicates at a density of 1×10^5^/well in a 6-well plate, and the number of colonies was counted 14 days later. Histogram (top) shows the mean ± S.E. of triplicate values from three independent experiments. Unpaired Student's *t*-test was used. (*) indicates p<0.05 value. Representative photographs of the foci are presented (bottom).

MTT assays revealed that the control MCF-7Vector cells continued to proliferate after 96 hours of starvation in serum free media, while control AU565Vector cells exhibited reduced growth (Fig. 5A). These differences might stem from a disparity in secreted cytokines/growth factors to the medium by these two cells lines [33]. In addition, Vav1 expression had opposing effects on proliferation in these two breast cell lines. At most time points during starvation, MCF-7Vav1 cells had a lower growth rate than the MCF-7Vector control cells. In contrast, AU565Vav1 cells had a higher growth rate than AU565Vector cells after 96 hours of starvation in serum free media (Fig. 5A).

Colony formation assays corroborated these MTT findings. When grown on soft agar, MCF-7Vav1 cells formed significantly smaller foci and a smaller number of foci than control cells (Fig. 5B). In contrast, AU565Vav1 cells formed significantly larger foci than control cells, while the number of foci remained the same (Fig. 5B).

### Vav1 Expression is Associated with Opposite Effects on Gene Expression in AU565 and MCF-7 Cells

**Figure 6:**
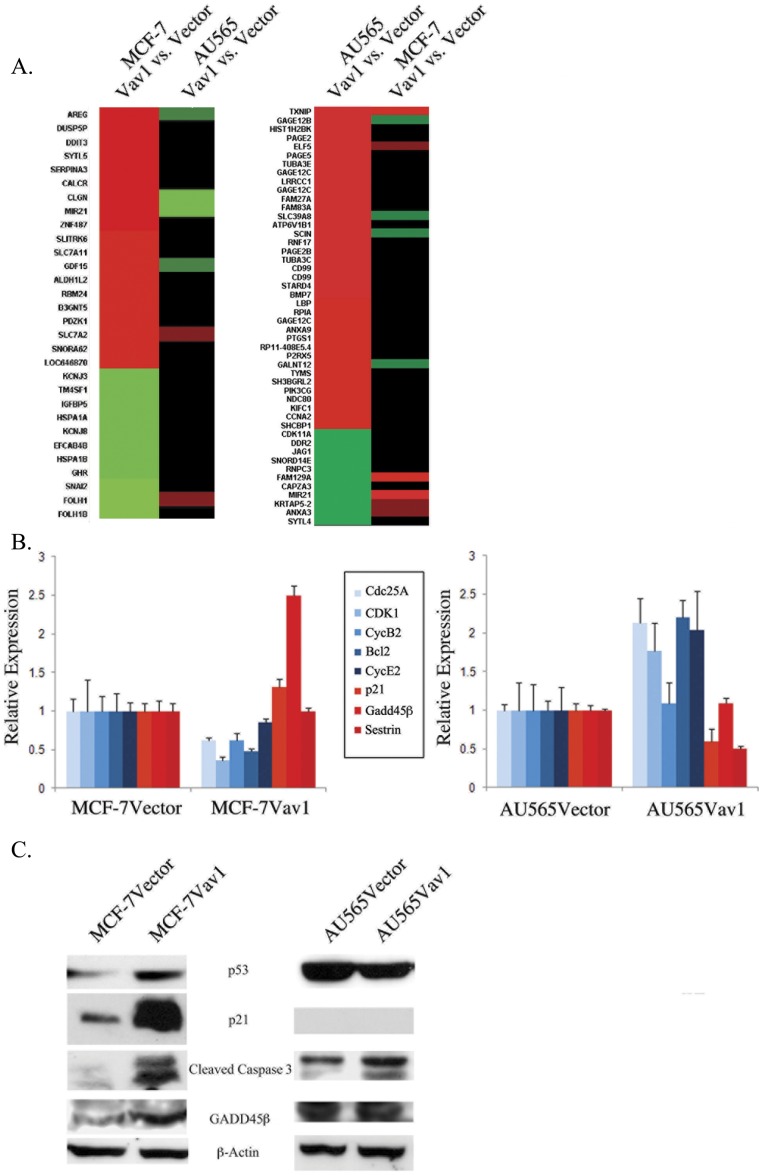
Vav1 expression leads to opposing changes in gene expression in MCF-7 and AU565 cells. (A) Affymetrix gene microarray of MCF-7Vector, MCF-7Vav1, and AU565Vector and AU565Vav1 cells was performed. For each line, gene expression in Vav1 expressing cells was compared with vector-transfected control cells. Left side of left panel, most significantly altered genes in MCF-7 cells. Right side of left panel, the same genes as expressed in AU565 cells. Left side of right panel, most significantly altered genes in AU565 cells. Right side of right panel, the same genes as expressed in MCF-7 cells. Each sample was composed of a mixture of three independent mRNA isolations. (B, C) Real Time PCR (b) or immunoblot (c) analysis of expression of selected apoptosis and proliferation-related genes in the two cell lines. Real Time PCR data show mean expression relative to expression in vector-transfected cells.

To better understand the contradictory effect of Vav1 expression on AU565 and MCF-7 cell lines, we performed global transcriptome assays. For each cell line, we compared gene expression in Vav1-transfected and vector-transfected cells. Interestingly, Vav1 expression caused significant alteration in gene expression patterns in both lines, but different genes were affected in each cell line. The most significantly altered genes in MCF-7Vav1 cells were not altered in AU565Vav1 cells and vice versa (Fig. 6A, both panels). A large group of apoptosis-related genes were elevated in MCF-7Vav1 cells, including Gadd45, Gadd153, Wee, Sestrins and Noxa. In contrast, a large number of proliferation-related genes were elevated in AU565Vav1 cells, including Cyclins A, B, D and E, Cdc25A, B and C, Cdk1 and 2 and PI3K. These observations were validated by quantitative RT-PCR of selected genes. Cdc25A, Cdk1, Cyclin B2 and Cyclin E were down-regulated in MCF-7Vav1 cells compared with MCF-7Vector cells and up-regulated in AU565Vav1 cells compared with AU565Vector cells. Conversely, p21, Gadd45β and Sestrin were up-regulated in MCF-7Vav1 cells compared with MCF-7Vector cells, but down-regulated in AU565Vav1 cells compared with control cells (Fig. 6B). Focusing on apoptosis-related genes, we compared p53, p21, cleaved caspase 3 and Gadd45β protein expression in Vav1 and control cells of both cell lines, and found all four proteins to be significantly elevated in MCF-7Vav1 cells and not altered or not present in AU565Vav1 cells (Fig. 6C).

### The Pro-apoptotic Effect of Vav1 on MCF-7 Cells is p53-dependant

**Figure 7:**
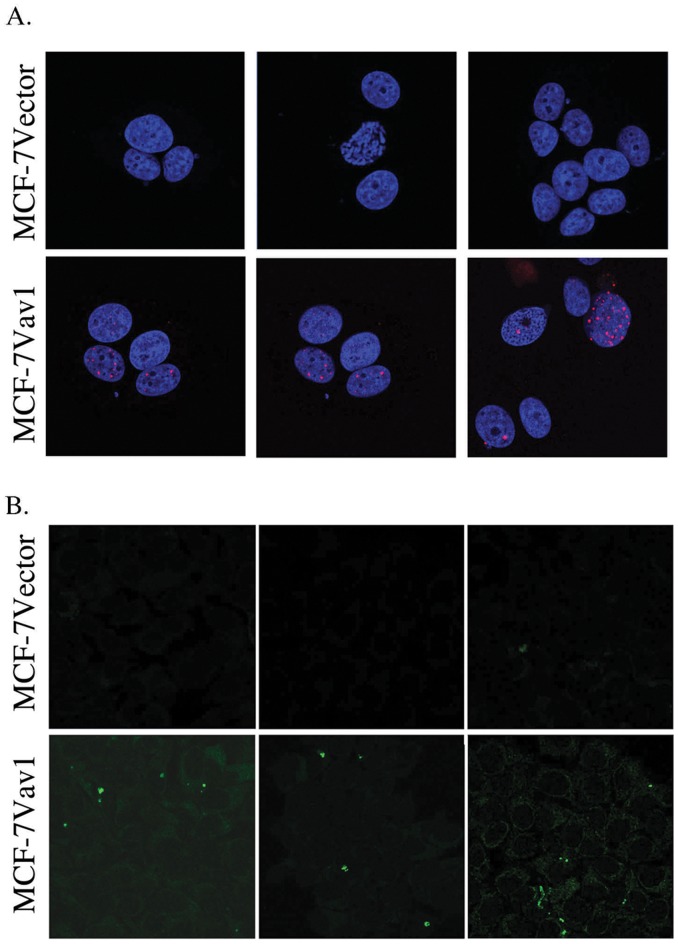
Vav1 pro-apoptotic effect in MCF-7 cells. (A) MCF-7Vector and MCF-7Vav1 were stained for detection of γ-H2AX foci. Three representative photographs of the foci from different areas are presented. (B) TUNEL assay of MCF-7Vector, MCF-7Vav1 cells was performed. Three representative photographs of cells from different areas are presented.

The MCF-7 breast cancer cell line expresses wild-type p53, while the AU565 breast cancer cell line carries a mutated p53 [34]. This raises the possibility that the opposite effects exerted by Vav1 in these cell lines are related to the function of p53 in these cells. To explore this possibility, we used two assays for apoptosis. First, we looked at γ-H2AX foci as a marker of DNA double-strand breaks, which occur where extensive apoptotic DNA fragmentation is present. There was a significant increase in the number of γ-H2AX foci in MCF-7Vav1 cells compared with MCF-7Vector cells (Fig. 7A). Similarly, TUNEL assay, which detects DNA fragmentation resulting from apoptotic signaling cascades, showed markedly elevated fragmentation in MCF-7Vav1 compared to control cells, consistent with a pro-apoptotic role of Vav1 in these cells (Fig. 7B). No such effect of Vav1 was seen in AU565 cells: neither γ-H2AX foci nor TUNEL fragmentation were elevated in AU565Vav1 cells compared with vector transfected cells (data not shown).

**Figure 8:**
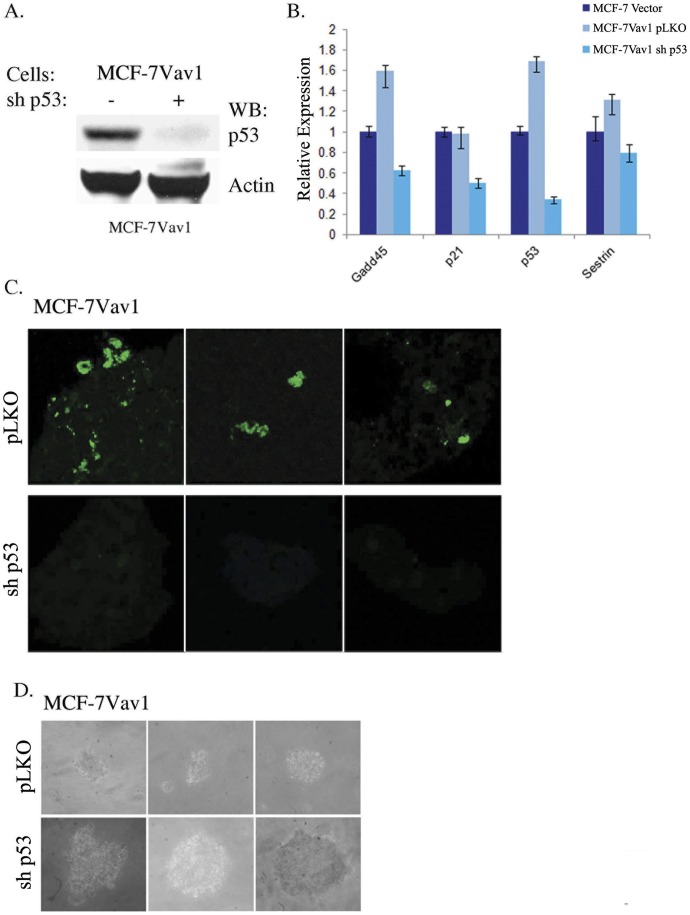
The pro-apoptotic effect of Vav1 on MCF-7 cells is p53-dependant. (A) p53 is silenced in MCF-7Vav1 cells following treatment with p53 shRNA. Cells were infected with viral vectors containing shRNA for p53 (shp53) or control (pLKO). Cell lysates were subjected to immunoblotting with anti-p53 antibody. Anti-actin was used as loading control. (B) Real Time PCR analysis of selected apoptosis-related genes. The analysis was performed on the following cells: MCF-7Vector or MCF-7Vav1 infected with control viral vector and MCF-7Vav1 infected with shp53. (C) TUNEL assay of MCF-7Vav1 infected with pLKO or shp53 was performed. Three representative photographs of the cells from different areas are presented. (D) MCF-7Vav1 infected with pLKO or shp53 were suspended in RPMI medium containing 0.3% agar and 10% calf serum, and plated onto a bottom layer containing 0.8% agar. Cells were plated in triplicates, at a density of 1×10^5^/well in a 6-well plate, and colonies were photographed 14 days later. Three independent experiments were analyzed, and representative photographs of cells from different areas are presented.

To verify the involvement of p53 in apoptosis in MCF-7Vav1 cells, we silenced p53 in MCF-7Vav1 cells using shRNA (shp53; Fig. 8A). Relative mRNA expression of Gadd45β, p21 and Sestrin was lower in p53-silenced MCF-7Vav1 cells than in cells treated with control shRNA (pLKO; Fig. 8B). The elevated fragmentation detected by TUNEL assay in MCF-7Vav1 cells was abolished in p53-silenced MCF-7Vav1 cells (Fig. 8C vs. 7B). Finally, p53-silenced cells showed an increase in the number and size of foci in the soft agar assay, erasing the Vav1 inhibitory effect (Fig. 8D and data not shown).

## Discussion

Our results indicate that wild-type Vav1 is expressed in a large percentage of human breast tumors. Furthermore, this aberrant expression of Vav1 was positively correlated with expression of ER and PR. ER plays a pivotal role in breast cancer development and progression, and is expressed in 75% of breast cancers [35]. ER expression is related to patient age and correlates with lower tumor grade, lower tumor proliferation, less frequent amplification of HER2 and concomitant loss of p53, positive expression of PR, less metastases, and slower rates of disease recurrence [36]. These clinical factors, along with ER expression itself, are used to guide treatment decisions in patients, especially those with metastatic disease. Clinicians use 21-gene and 70-gene profiles to classify ER-positive tumors according to their aggressiveness, risk of recurrence, and likelihood of benefiting from adjuvant endocrine or chemotherapy [37], [38]. Based on our results, Vav1 can be included in the existing gene profiles, especially in light of its unexpected expression in a breast cancer cell line such as MCF-7 following treatment with estradiol (Fig. S1); however its prognostic value remains to be determined. Two previous attempts to associate Vav1 with human breast cancer used smaller cohorts of breast cancer specimens and a very small number of human breast cancer cell lines and are less informative and therefore were inconclusive [39], [40].

It is possible that in several of the breast cancer cell lines cbl-c expression is elevated, leading to Vav1 ubiquitination. A number of studies have suggested a potential role for Cbl in regulating Vav1 in the hematopoietic system. Vav1 was shown to interact with phosphorylated Cbl through its SH2 region in both thymocytes and peripheral T cells following stimulation through the TCR [41]. Notably, T cells from Cbl-b-deficient mice showed enhanced Vav1 phosphorylation and TCR clustering upon TCR stimulation [42], [43], and Cbl-b deficiency restored the defective TCR clustering observed in Vav^−/−^ T cells [44]. It has also been demonstrated that Cbl functions as an ubiquitin ligase toward Vav1 and that this activity allows Cbl to negatively regulate Vav1-mediated signaling [26]. Lastly, the requirement for Vav1 was completely eliminated in Vav1^−/−^Cbl^−/−^ mice, with full normalization of T cell development [27]. Our results provide the first evidence for regulation of Vav1 expression by Cbl-c in non-hematopoietic cells, and specifically, in cancer cells.

In non-hematopoietic cells, it is likely that aberrantly expressed Vav1 is activated by various membrane receptors and triggers signaling cascades that result in cytoskeletal reorganization and transcription. Recent studies in pancreatic cancer [6] and lung cancer [7] cells that express Vav1 showed that Vav1 functions as a GEF for Rac1 GTPase following EGF stimulation and that this activity is critical for its function. When we expressed Vav1 in AU565 cells, we indeed observed remarkable Rac1 activation and changes in cytoskeleton organization including lamellipodia formation, pointing to increased potential for motility. However, expression of Vav1 in MCF-7 cells induced different cytoskeletal changes. Interestingly, no Rac1 activation was observed in this case, suggesting that other signaling cascades can mediate Vav1-induced cytoskeleton reorganization.

We show that Vav1 is tyrosine phosphorylated in AU565Vav1 and MCF-7Vav1 cells in response to EGF and CSF1 stimulation respectively. The time course of Vav1 phosphorylation differed in AU565Vav1 and MCF-7Vav1 cells, again suggesting that distinct signaling cascades are activated in these cell lines. Furthermore, ERK phosphorylation was significantly enhanced in response to cell stimulation and Vav1 phosphorylation in MCF-7Vav1 cells, but not in AU565Vav1 cells, suggesting the proliferative effect of Vav1 may be mediated by an ERK signaling cascade in AU565 cells.

Recent data demonstrated that Vav1 can stimulate secretion of autocrine ligands that can activate the EGFR, another mechanism by which Vav1 might contribute to tumorigenicity. Depletion of Vav1 in lung cancer cells decreased expression of TGFα, an autocrine growth factor that activates these cells [7]. In the human mammary epithelial cell line MCF-10A, expression of a constitutively active form of Vav1 promoted migration and induced morphological changes [45]. This increased migration was dependent on Vav1 GEF activity, which stimulated the Rac1-Pak pathway, and on secretion of an autocrine EGF receptor ligand. These studies support the existence of feed-forward loops in which Vav1 regulates secretion of autocrine ligands, leading to receptor stimulation and subsequent increases in Vav1 activation. The additional stimulatory input provided by Vav1 signaling in cells where it is aberrantly expressed may overwhelm control mechanisms and tip the scales in favor of transformation.

Our soft agar and MTT assays showed opposing phenotypes of AU565 and MCF-7 cells ectopically expressing Vav1. In AU565Vav1 cells we observed an increase in foci number and size, indicative of a higher proliferation rate in comparison to AU565Vector cells. We observed the opposite in MCF-7Vav1 cells, which formed smaller foci than MCF-7Vector cells. These surprising results raised the possibility that the expression of Vav1 affects gene expression in a different manner in these cell lines. Indeed, Vav1 leads to an increase in expression of pro-proliferation genes in AU565, while pro-apoptosis genes are elevated in MCF-7 cells.

The anti-apoptotic effect of Vav1 has been shown in cancers of hematopoietic origin. *In vitro* knockdown of Vav1 in anaplastic large cell lymphoma was sufficient to cause cell cycle arrest and apoptosis of these cells [46]. In the HL-60 and NB4 promyelocytic cell lines, down-regulation of Vav1 affected expression of a number of cell cycle/apoptosis-related proteins [47]. Lastly, Vav1 was found to protect Jurkat T cells from Fas-mediated apoptosis by promoting Bcl-2 transcription through its GEF activity toward Rac2 [48]. Other studies have pointed to a pro-apoptotic role for Vav1 in hematopoietic cells. For instance, during negative selection, Vav1 promotes antigen-induced thymocyte apoptosis, and inhibitors of the actin cytoskeleton or protein kinase C (PKC) reverse the effect [49]. In activated CD4^+^ T cells, the Vav1-Rac pathway is a critical component of TCR-induced cell death [50]. Vav1 was also shown to mediate apoptosis in L-MAT, a human lymphoblastic T cell line [51]. In macrophages, the engulfment of apoptotic cells requires the activation of Vav1/Rac1 and subsequent actin polymerization to form the phagocytic cup [52].

We demonstrate here for the first time that Vav1 can influence apoptosis in non-hematopoietic cancer cells. The normal cellular response to oncogenic stress requires the tumor suppressor protein p53. Nevertheless, the mechanisms linking oncogene activation to p53 induction have remained controversial. Evidence from studies of early-stage human tumors and animal models suggest that oncogene-induced replication stress activates a DNA damage response (DDR), which in turn activates p53 [53]–[55]. Using γH2AX and TUNEL assays, we observed significant DDR in our MCF-7Vav1 cells, as well as a remarkable increase in several apoptosis-related proteins. We also demonstrated that the apoptotic phenotype of MCF-7Vav1 cells is p53-dependant. Several oncogenes and tumor suppressor genes have been shown to have dual behavior in cancer, dependent on the cellular environment. One such example is the transcription factor NF-κB. The role of the NF-κB signaling pathway in cancer is complex. While in some cancers, NF-κB is oncogenic, and can serve as an excellent target for tumor therapy, there is evidence it can also suppress tumorigenesis [56]. Another example of a protein with a dual role is Yap, a small protein that binds to many transcription factors and modulates their activity. Yap increases the pro-apoptotic function of p73 following DNA damage, and therefore its activity favors tumor-suppression. However, other studies have recently shown a role for Yap in cell differentiation, cell transformation and in the regulation of organ size [57].

Whether Vav1 can play a dual role as a pro- or an anti-apoptotic protein in cancer cells of non-hematopoietic origin has never been tested directly, yet several studies point to such roles in hematopoietic cells. While Vav1 was shown to protect Jurkat T cells from Fas-mediated apoptosis by promoting Bcl-2 transcription through its GEF activity [46], Gu *et al.,* demonstrated that oncogenic Vav1, which is constitutively active as a GEF, induces Rac-dependent apoptosis via inhibition of Bcl-2 family proteins and collaborates with p53 deficiency to promote hematopoietic progenitor cell proliferation [58]. Thus, it is conceivable that in non-hematopoietic cancer cells wild-type Vav1 might function in a similar fashion to oncogenic Vav1 in hematopoietic cells due to its constitutive activation by various aberrantly functional signaling cascades. Moreover, its activity could depend on additional genetic aberrations, such as the p53 pathway.

The fact that Vav1 is shown by us in this study to have opposite effects when expressed in two breast cancer cell lines, MCF-7 and AU565, clearly highlights the importance of the cellular environment on Vav1 function. Similarly, CKIα was recently shown to be tumor suppressive when p53 is inactive. Combined ablation of CKIα and p53 triggered high-grade dysplasia with extensive proliferation [59].

NF-κB, Yap and CKIα represent three major developmental pathways (NF-κB, Hippo and Wnt signaling, respectively) that can lead to transformation when aberrantly expressed. Our results highlight a similar role for Vav1, an important player in its own signaling cascade in thymocytes, which contributes to cancer development when aberrantly expressed in the breast. In addition, our results indicate that the effect of ectopic Vav1 expression is highly dependent on other cellular factors, including p53 availability.

## Supporting Information

Figure S1 **Vav1 expression in MCF-7 cells following treatment with estradiol.** MCF-7 cells were treated for 48 hr with 0, 10 and 20 nM of estrodiol (SIGMA). cDNA was subjected to RT-PCR using Vav1 primers. Actin was used as a loading control.
(TIF)

Table S1 **Primers used for Real-Time PCR and shRNA sequences.** This table details the sequences of primers used for Real-Time PCR performed. Also, included are the sequences used for shRNA.
(DOC)

Table S2 **Antibodies used for Immunoprecipitation, Immunoblotting, Immunohistochemistry and Immunofluorescence.** The antibodies for western blotting, immunoprecipitation, immunohistochemistry and immunofluorescence used in the study are detailed, including the source for their purchase.
(DOC)

Table S3 **Vav1 expression in breast cancer tissue array.** The table details the various cancer tissues used in the study including their receptor expression (ER, PR, and HER2 from – to +++) and cancer staging (TNM) according to the manufecturere's information. Also, included is the level of Vav1 protein expression calculated as detailed in the Material and Methods section.
(XLS)

Table S4 **Vav1 (mRNA and protein) and Cbl-c (mRNA) expression in various breast cancer cell lines.** The mRNA and protein expression level of Vav1 and mRNA expression of Cbl-c as assessed in our experiments (−; +/−; ++) in various human breast cancer cell lines used in our experiments.
(XLS)
