# Supplementary figures and images for: Vav1 Fine Tunes p53 Control of Apoptosis versus Proliferation in Breast Cancer
Source: PLoS One. 2013 Jan 14;8(1):e54321. doi: 10.1371/journal.pone.0054321 (PMC3544807; doi:10.1371/journal.pone.0054321)

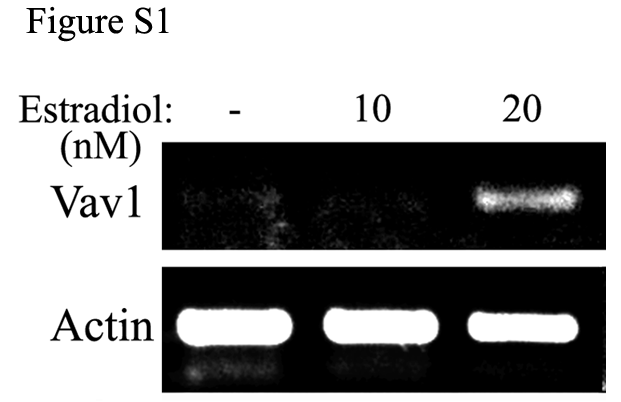

Supplement: Figure S1 — Vav1 expression in MCF-7 cells following treatment with estradiol. MCF-7 cells were treated for 48 hr with 0, 10 and 20 nM of estrodiol (SIGMA). cDNA was subjected to RT-PCR using Vav1 primers. Actin was used as a loading control. (TIF) [file pone.0054321.s001.tif]
