# Supplementary material for: Vav1 Fine Tunes p53 Control of Apoptosis versus Proliferation in Breast Cancer
Source: PLoS One. 2013 Jan 14;8(1):e54321. doi: 10.1371/journal.pone.0054321 (PMC3544807; doi:10.1371/journal.pone.0054321)
Supplement: Table S1 — Primers used for Real-Time PCR and shRNA sequences. This table details the sequences of primers used for Real-Time PCR performed. Also, included are the sequences used for shRNA. (DOC) [file pone.0054321.s002.doc]

Table S1: Primers used for Real-Time PCR and shRNA sequences

| shRNA sequences | Reverse primer | Forward primer | Gene |
| --- | --- | --- | --- |
| - | 5'-TTGAAGAGGCTTATGGTTTGCTT-3' | 5'-GGTGCGGTTGTGTCAGAAC-3' | c-Cbl |
|  | 5'-CTCTAAGGCTCTCTTCACCTCT-3' | 5'-ACCAGCCAGACCCCCTAAA-3' | Cbl-b |
|  | 5'-ACAGGGGTTTGTTGGCAGG-3' | 5'-GTGGGCCATCGGCTATGTG-3' | Cbl-c |
|  | 5'-GAGTGCAGGCAGCCACGAGA-3' | 5'-CCCTACCTCAGAAGCTGTTGGGA-3' | CDC25A |
|  | 5'-ACAAAACACAATCCCCTGTAGG-3' | 5'-TCCCTCCTGGTCAGTACATGG-3' | Cdk1 |
|  | 5'-TGTCCTCGATTTTGCAGAGCA-3' | 5'-AAGCTCAGAACACCAAAGTTCC-3' | CycB2 |
|  | 5'-CTTGGGCGAGAGCTGTCATC-3' | 5'-GCCCCTGCTATGGTTTAGAGC-3' | Bcl2 |
|  | 5'-GCCAGGAGATGATTGTTACAGG-3' | 5'-TCCAAGAGTTTGCTTACGTCAC-3' | cycE2 |
|  | 5'-GGCGTTTGGAGTGGTAGAAATCT-3' | 5'-CTGGAGACTCTCAGGGTCGAA-3' | P21 |
|  | 5'-CCCGGCAAAAACAAATAAGTTG-3' | 5'-TGCGTGCTGGTGACGAAT-3' | Gadd45α |
|  | 5'-CGCCCAGAGGACATCAGTG-3' | 5'-AAGGACTACCTGCGGTCCG-3' | Sestrin |
|  | 5'-ACCAAATCCGTTGACTCCGACCTT-3' | 5'-TCGACAGTCAGCCGCATCTTCTTT-3' | GAPDH |
| CCGGCCCAGAAATGCCCAGCCGAAACTCGAGTTTCGGCTGGGCATTTCTGGGTTTTT |  |  | CBLC |
| CCGGCGTCGAGGTCAAGCACATTAACTCGAGTTAATGTGCTTGACCTCGACGTTTTTG |  |  | VAV1 |
| CCGGCGGCGCACAGAGGAAGAGAATCTCGAGATTCTCTTCCTCTGTGCGCCGTTTTT |  |  | TP53 |
