# Supplementary material for: Vav1 Fine Tunes p53 Control of Apoptosis versus Proliferation in Breast Cancer
Source: PLoS One. 2013 Jan 14;8(1):e54321. doi: 10.1371/journal.pone.0054321 (PMC3544807; doi:10.1371/journal.pone.0054321)
Supplement: Table S2 — Antibodies used for Immunoprecipitation, Immunoblotting, Immunohistochemistry and Immunofluorescence. The antibodies for western blotting, immunoprecipitation, immunohistochemistry and immunofluorescence used in the study are detailed, including the source for their purchase. (DOC) [file pone.0054321.s003.doc]

*Table S2: Antibodies used for Immunoprecipitation, Immunoblotting, Immunohistochemistry and Immunofluorescence*

| Name of Antibodies & Purpose | Manufacturer information |
| --- | --- |
| Monoclonal anti-Vav1 (for western blotting, immunohistochemistry and immunofluorescence | Upstate Biotechnology, NY, USA; #05-219 |
| anti-Rac1 | Upstate Biotechnology, NY, USA; #05-389 |
| anti-phosphotyrosine | Millipore, MA, USA; #05-321 |
| anti-ERK | Millipore, MA, USA; #06-182 |
| anti-γh2ax | Millipore, MA, USA; #AB10022 |
| anti-pERK | Cell Signaling, MA, USA; #9106S |
| anti-cleaved caspase-3 | Cell Signaling, MA, USA; #9661L |
| anti-Flag | Sigma, Rehovot, Israel; #087K6002 |
| anti-p21 | Santa Cruz, USA; #SC6246 |
| anti-Gadd45β | Santa Cruz, USA; #SC8775 |
| anti-actin | Santa Cruz, USA; #SC130656 |
| rabbit polyclonal anti-Vav1 antibodies for immunoprecipitation | Katzav S, et al. (1991) *Mol. Cell. Biol.* 11: 1912-1920 |
| anti-human p53 | DO1, 1801 mixed hybridomas |
| AlexaFluor-647 anti-mouse IgG | Molecular probes, USA; #A21235 |
| Alexa fluor-546 Phalloidin staining for actin cytoskeleton | Molecular probes, USA; #A22283 |
